# HDS-Net: Achieving fine-grained skin lesion segmentation using hybrid encoding and dynamic sparse attention

**DOI:** 10.1371/journal.pone.0299392

**Published:** 2024-03-21

**Authors:** You Xue, Xinya Chen, Pei Liu, Xiaoyi Lv

**Affiliations:** 1 College of Information Science and Engineering, Xinjiang University, Urumqi, China; 2 College of Software, Xinjiang University, Urumqi, China; GIET University, INDIA

## Abstract

Skin cancer is one of the most common malignant tumors worldwide, and early detection is crucial for improving its cure rate. In the field of medical imaging, accurate segmentation of lesion areas within skin images is essential for precise diagnosis and effective treatment. Due to the capacity of deep learning models to conduct adaptive feature learning through end-to-end training, they have been widely applied in medical image segmentation tasks. However, challenges such as boundary ambiguity between normal skin and lesion areas, significant variations in the size and shape of lesion areas, and different types of lesions in different samples pose significant obstacles to skin lesion segmentation. Therefore, this study introduces a novel network model called HDS-Net (Hybrid Dynamic Sparse Network), aiming to address the challenges of boundary ambiguity and variations in lesion areas in skin image segmentation. Specifically, the proposed hybrid encoder can effectively extract local feature information and integrate it with global features. Additionally, a dynamic sparse attention mechanism is introduced, mitigating the impact of irrelevant redundancies on segmentation performance by precisely controlling the sparsity ratio. Experimental results on multiple public datasets demonstrate a significant improvement in Dice coefficients, reaching 0.914, 0.857, and 0.898, respectively.

## 1. Introduction

Skin cancer is among the most common malignant tumors worldwide, and early detection is important to improve its cure rate [1]. In the field of medical imaging, precise segmentation of lesion areas in skin images holds significant value, enabling physicians to swiftly diagnose and effectively treat patients [2]. However, this task is confronted with several challenges. The boundaries between normal skin areas and lesion areas in skin images are often ambiguous, with notable variations in the size and shape of the lesion areas. Moreover, the types of lesions vary greatly among different samples. Fortunately, the rapid evolution of deep learning technology has paved the way for innovative solutions to address these challenges [3–5].

In the task of skin lesion segmentation, deep learning models have been widely applied. They can learn highly abstract features from image data, enabling accurate segmentation of different types of skin lesion areas [6–10]. However, applying deep learning techniques to skin lesion segmentation is not a straightforward task. The traditional U-Net architecture [11] is a common image segmentation framework known for its excellent feature extraction capabilities, primarily relying on multi-level cascaded convolutions. All convolutional layers repetitively extract regions of interest (ROI) and then make predictions, similar to how it processes cardiac MRI [12–14] and cardiac CT [15] images. However, this method merely iteratively extracts local features. In skin lesion images, not only do the shape and size of lesion areas vary significantly, but the boundaries between normal and lesion tissues are also blurred. Therefore, the repeated use of cascaded convolutional layers cannot efficiently identify the lesion areas.

In recent years, many variants of U-shaped network structures have emerged [16–19], such as U-Net+ [20], Attention U-Net [21], U-Net+++ [22],etc. However, due to the traditional convolutional approach lacking in extracting global information effectively, it cannot satisfactorily address the skin lesion area segmentation problem. Recently, networks based on the Transformer have begun to be applied to medical image segmentation tasks [23–25], providing a new perspective for tackling skin lesion segmentation issues [26]. The Transformer structure is renowned for its global modeling ability using self-attention mechanisms, but compared to traditional convolutions, it is slightly lacking in extracting local information [27, 28]. This discovery inspired us to propose a novel network HDS-Net.

The size of skin lesion areas often exhibits significant variations, and the boundaries between normal skin and disease regions can be relatively ambiguous due to complex background information (such as hair) interference. Additionally, different samples may present diverse types of lesions [1]. These factors make it challenging for traditional convolutional methods to accurately segment lesion areas. To address this issue, this paper introduces a hybrid encoder. During the feature extraction process, this encoder incorporates a dynamic sparse attention mechanism, which highlights salient features relevant to skin lesion segmentation while suppressing irrelevant region features. This enhances the recognition accuracy and sensitivity towards regions of interest (ROI). Furthermore, two learnable memory units are employed to establish potential connections between different samples, thereby strengthening the network’s ability to recognize various types of lesions.

To validate the effectiveness of the proposed approach, experiments and evaluations were conducted on three commonly used skin datasets (ISIC2016, ISIC 2017, and ISIC2018) [29–31] The results demonstrate a significant improvement in segmentation accuracy with HDS-NET. The primary contributions of this study are as follows:

1. Proposing a hybrid encoder that effectively extracts local information while establishing connections with global information.
2. Designing a dynamic sparse attention mechanism that enhances the network’s focus on relevant information by controlling the sparsity ratio of the sparse matrix, thereby suppressing redundant information.

## 2. Related work

U-Net, as a classic model in the field of image segmentation, has demonstrated outstanding performance in numerous segmentation tasks. However, the stacked convolutional operations in its encoding and decoding stages may lead to information loss. To overcome these limitations, researchers have proposed various improved models such as MAS-UNet [17], U2Net [32], Multi-UNet [33], and others. For instance, U-Net++ enhances the capture of multiscale information by introducing cross-scale connections. UNet+++ further employs the U-Net++ structure in each branch to enhance the network’s ability to capture complex structures and multiscale information. However, the performance of these methods often highly depends on the quality and diversity of training data. In real-world scenarios, collecting suitable training data for skin lesion analysis remains challenging. Unlike the traditional U-Net family, Attention U-Net introduces self-attention mechanisms and recombination modules, achieving adaptive feature selection and diverse feature representation, thereby improving the segmentation performance and generalization ability of the model. However, skin lesion images exhibit diverse shapes, and the boundaries between normal skin and lesion areas are often blurry. Furthermore, there are feature differences between the center and edges of lesion regions. The repeated convolutional structures and downsampling processes used in traditional convolutional networks may result in the loss of foreground information when extracting lesion-related information, making the boundaries of the segmentation image fuzzy or discontinuous. Despite the efforts made by the traditional U-Net improvement family in this regard, they still struggle to meet the demands of skin lesion segmentation.

Due to the limitations of convolutional operations, it is challenging to learn global information and long-distance relationships between pixels. As a result, some researchers have attempted to use techniques such as dilated convolutions [34] and image pyramids [35]. However, these methods still have certain limitations in establishing global connections, as they overlook the contextual relationships within samples and the potential connections between different samples.

In recent years, the impressive performance of Transformers has garnered significant attention in the computer vision field. Although Transformers were initially designed for machine translation tasks, their exceptional global modeling capabilities have led many researchers to apply them to computer vision tasks to address the challenges of establishing global connections that convolutional operations struggle with. The Vision Transformer (ViT) [36] was the first successful attempt to introduce Transformers into the computer vision domain. While initially designed for image classification tasks, the outstanding performance of Transformers inspired researchers to apply them to medical image segmentation tasks, aiming to establish long-distance connections and extract complex structural information more effectively. TransUNet [37] introduced a Transformer branch on top of the traditional convolutional U-Net, making full use of the U-Net’s encoder-decoder structure. This approach effectively extracts local features while establishing connections between global features. Swin-UNet [38] took a step further by completely replacing convolutional operations with Transformer operations. It maintains the integrity of feature details through skip connections while retaining the model’s efficiency and the ability to learn long-distance dependencies.

In summary, skin lesion areas exhibit significant variations in size, and the boundaries between normal and lesioned skin regions are often blurry. Convolutional operations excel at extracting local information but struggle to efficiently establish long-distance connections between pixels. Transformers, on the other hand, efficiently learn global features but have relatively weaker capabilities in modeling local information. Building on previous research, this paper introduces a U-shaped network architecture and incorporates a dynamic sparse attention mechanism. This approach not only facilitates long-distance modeling within samples but also enhances the model’s generalization ability, effectively addressing these challenges.

## 3. Method

**Fig 1:**
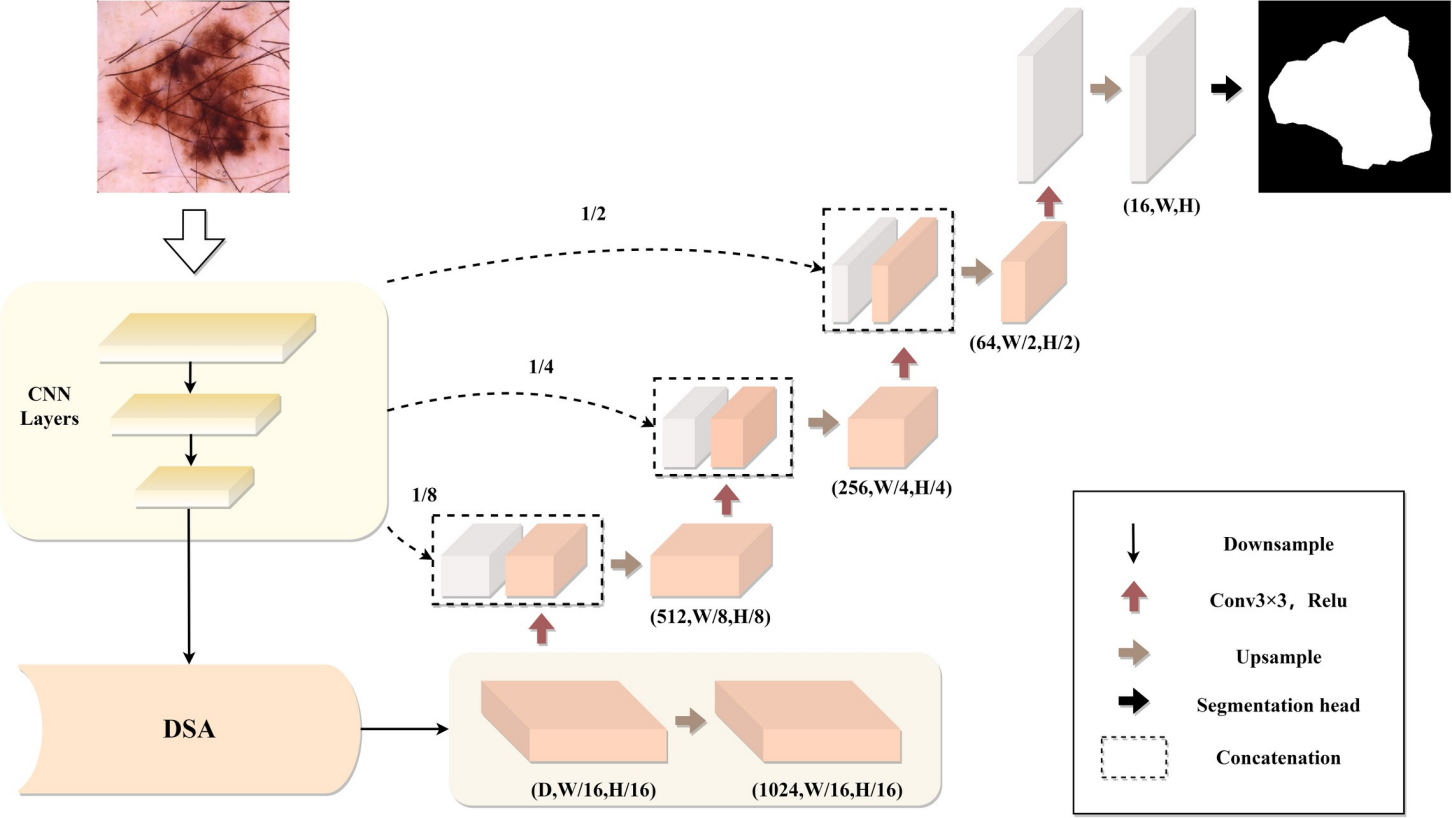
The structure of the HDS-Net.

The network architecture proposed in this paper is illustrated in Fig 1. The encoder consists of CNN and external attention modules, aiming to better extract feature information, while the decoder restores feature maps through transpose convolutions. Skip connections play a critical role between the encoder and decoder. In the CNN section, skip connections are directly concatenated with their corresponding decoders when the feature map size is reduced to 1/8, 1/4, and 1/2, respectively, aiding in preventing information loss caused by downsampling operations. Next, we will provide a detailed description of the structure and functionality of each module.

### 3.1 CNN layers

**Fig 2:**
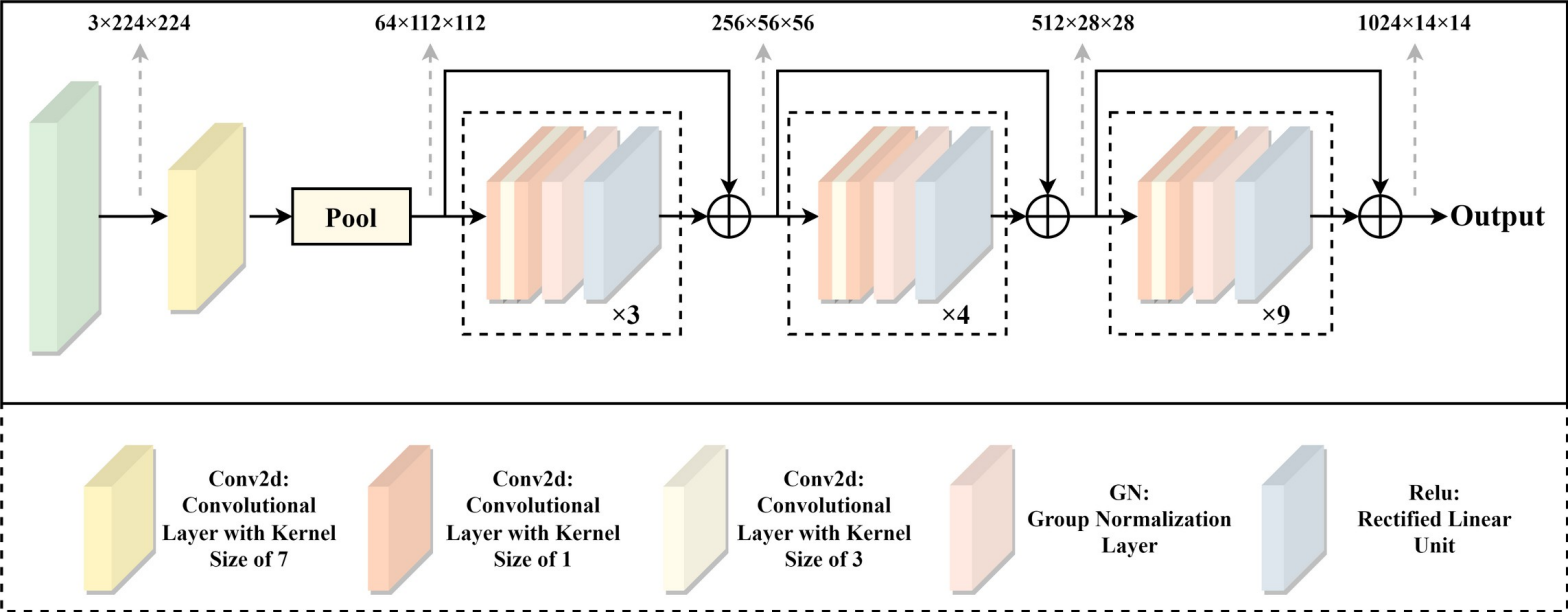
The structure of the CNN.

The encoder of HDS-Net is divided into two parts: CNN layers and external attention modules. Firstly, the CNN Layers section utilizes an encoder structure similar to ResNet50 [39] to extract shallow features. Additionally, due to the potential for significant experimental bias introduced by large input scales, this study adopts group normalization to effectively mitigate the impact of batch input scale on the experimental results. The specific architecture is illustrated in Fig 2.

### 3.2 DSA layers

Self-attention possesses powerful global modeling capabilities, aiming to capture long-range dependencies by calculating the dependency relationships between elements within the features. In skin segmentation tasks, given the presence of various lesion types and complex background scenarios, a critical challenge is to enable the model to better extract local information while establishing connections between global information. Simultaneously, it is essential to minimize complex background information to accurately segment foreground information. The proposed Dynamic Sparse Attention (DSA) module in this paper constitutes the second part of the hybrid encoder. In this module, the feature map first undergoes 1*1 convolutions for local information extraction. Subsequently, dimension transformation is performed, converting the original feature map of size Batchize(B) * Channel(C) * Height(H) * Widht(W) to B * C * H × W. The transformed feature map is then input into the DSA module. After passing through the DSA module, the feature map is restored to its original dimensions using the same operations. This process effectively extracts local information from the features while enhancing connections for global information. The main transformation process is illustrated in Formula 1 and 2. ***X*** represents the input feature map, ***DSA*** represents the Dynamic Sparse Attention module, ***Out*** represents the output after passing through the DSA module, and ***conv*** is a 1x1 convolutional kernel.


f(X)=reshape[conv(X)] (1)

out=f[DSA[f(X)]] (2)

***DSA*** is used to control the sparsity of feature maps by manipulating the probability values of the Bernoulli-masked matrix [40]. Since the skin segmentation task is essentially a binary classification task, requiring the segmentation of foreground and background information, a Bernoulli binomial distribution is used to randomly generate sparse matrix feature maps as shown in the formula 3. The introduction of randomness and sparsity during the training process adds more possibilities, preventing the model from getting trapped in local minima. This significantly enhances the model’s generalization ability and robustness. Additionally, in this study, element-wise multiplication is employed instead of traditional matrix multiplication, placing a stronger emphasis on extracting local information while establishing long-term connections. The specific computation process is depicted in Formula 4 and Formula 5, f(k) represents the probability values of generating 0 and 1 in the matrix. p represents the probability of the value being 1 in the matrix, while 1-p represents the probability of the value being 0 in the matrix, k represents the elements in the matrix. ***α***_***i***_ represents the sparse attention scores controlled by the Bernoulli matrix, ***v***_***i***_ represents the learned memory units from global image features, memory units, or contextual information, and ***O***_***i***_ represents the features extracted through the DSA module. ***L*** denotes Linear, ***F_in_*** represents input features, F^*^ signifies external memory units, and ***F***_***out***_ denotes output features. ***x*** represents the probability values generating the Bernoulli matrix from the binomial distribution.


$$\text{f}(k)=\left\{\begin{array}{c} \;p\;\;\;\;\;\;\;\;\;\;\;\;\;\;\;\;\text{if}\;k=1\\ 1-p\;\;\;\;\;\;\;\;\;\;\text{if}\;k=0 \end{array}\right.$$ (3)

$$O_{i}=\alpha_{i}\times v_{i}$$ (4)

$$F_{out}=\left[softmax\left[L\left(F_{1}^{*}\right)\times L\left(F_{in}\right)\right]\times Bernoulli(x)\right]\times L\left(F_{2}^{*}\right)$$ (5)

**Fig 3:**
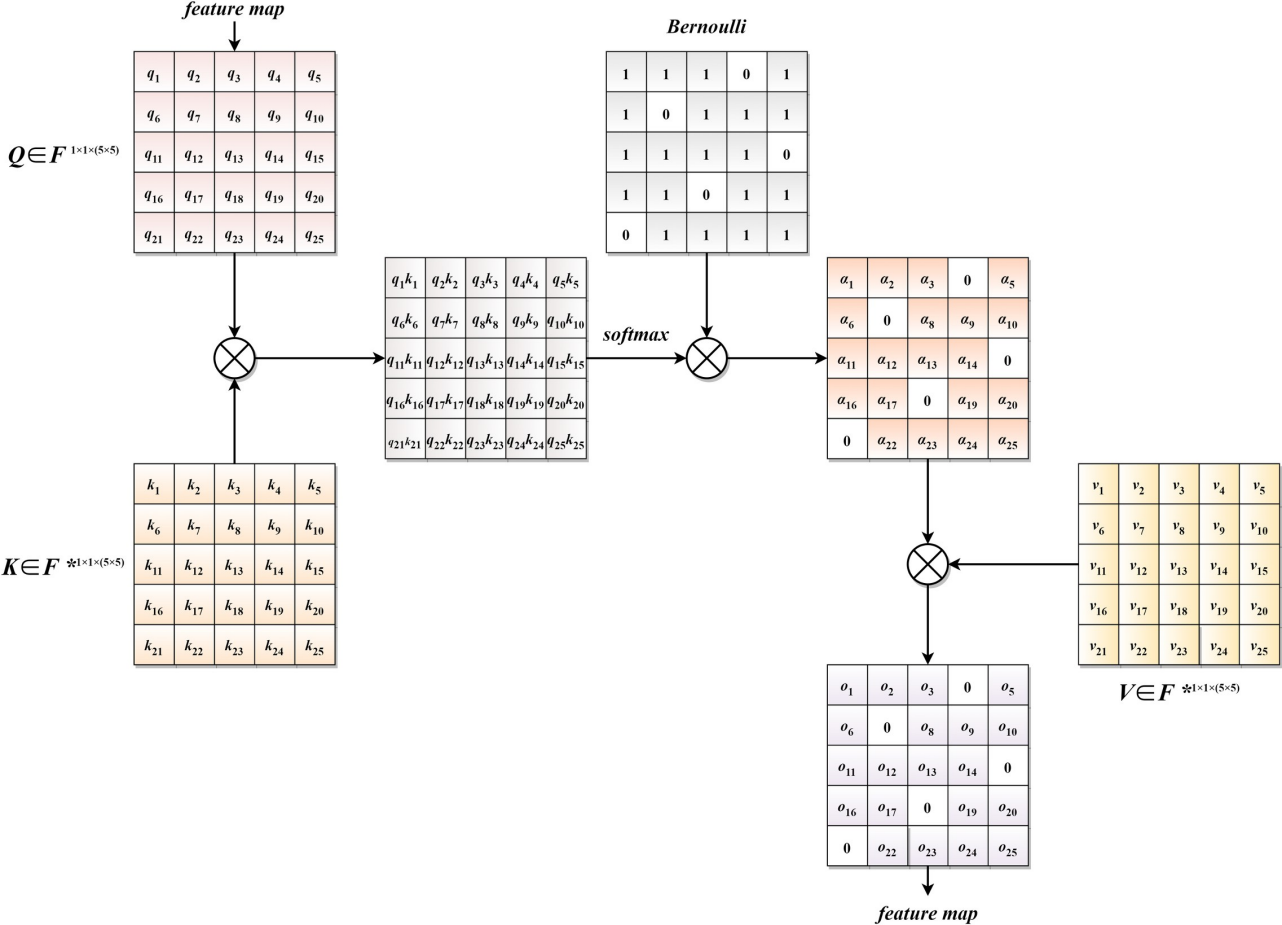
Schematic diagram of DSA computation.

The dynamic sparse attention mechanism is illustrated in the accompanying Fig 3. Assuming the convolutional feature map **Q** has dimensions 1*1*5×5, and two learnable memory units ***K*** and ***V*** are not directly derived from image features but instead come from external information, typically global image features, memory units, or contextual information. ***K***, ***V***, and the feature map ***Q*** maintain the same shape. Firstly, the feature map ***Q*** is element-wise multiplied with the learnable memory unit ***K***, generating an attention score matrix ***M*** to measure the relationship between ***Q*** and ***K***. Subsequently, we normalize ***M*** and element-wise multiply it with a Bernoulli distribution-random masked matrix, adding randomness and sparsity while allowing the network to better extract local information features. The resulting matrix is then multiplied with ***V*** to obtain a new feature map. This feature map not only associates relationships within the sample but also establishes correlations between elements from different samples.

In our task of skin lesion segmentation, the introduction of the dynamic sparse attention module aims to enhance the model’s ability to model the correlations between different skin lesion samples. By dynamically adjusting weights in the attention mechanism, the model can selectively focus on key lesion areas in the image, thus gaining a more comprehensive understanding of the global contextual information in skin images. This dynamism helps the model better adapt to different lesion types and image structures, ultimately improving overall performance in skin lesion segmentation. Element-wise multiplication, particularly direct multiplication operations between elements, holds special significance in skin lesion segmentation. By emphasizing element-wise products, the model can more sensitively capture the correlations around the lesion boundaries and their surrounding areas, aiding in more accurate segmentation and localization of skin lesions. In medical images, this local correlation modeling is crucial for capturing subtle lesion, edge, and structural changes. The use of a random Bernoulli masking matrix performs exceptionally well in skin lesion segmentation tasks. By introducing randomness, the masking matrix effectively suppresses complex background information unrelated to skin lesions, allowing the model to focus more on the features of lesion regions. This introduction of randomness enables it to better adapt to different lesion morphologies and background conditions.

By integrating these mechanisms, our model excels in skin lesion segmentation tasks, capturing lesion information in skin images at both global and local scales. These advanced correlation modeling techniques provide powerful tools for our research, leading to significant progress in the field of dermatological image analysis. They offer robust support for improving the accuracy and robustness of skin lesion segmentation.

## 4. Experiment

### 4.1 Dataset

The HDS-Net proposed in this paper was evaluated on three different skin lesion datasets, namely, ISIC2016, ISIC2017, and ISIC2018. These datasets comprise a significant number of skin images of common pigmentary skin lesions sourced from various races, skin tones, regions, and journals. The data sets were randomly divided into training and testing sets in 8:2. As shown in Table 1. Data set address: https://challenge.isic-archive.com/data.

**Table 1:** Dataset split settings.

Dateset	Train	Test
ISIC2016	1279	379
ISIC2017	2150	600
ISIC2018	2074	520

### 4.2 Loss function

To achieve optimal training outcomes and accurate segmentation results, consideration was given to the high similarity between medical image samples, significant differences between lesion and normal regions within samples, as well as the issue of class imbalance in the lesion segmentation areas. In this study, a composite loss function was utilized, which combines the Dice coefficient with binary cross-entropy. The Dice loss function was employed to address the class imbalance issue, while the binary cross-entropy loss function was used to mitigate the instability in backpropagation that may arise from using the Dice loss function. The loss function consists of two components: the Dice coefficient loss as the first part and the binary cross-entropy loss as the second part. The expression of the loss function is shown in Formula 5.


$$L_{\text{Loss}}=L_{\text{Dice}}+L_{\text{Cross-Entroy}\;\text{Loss}}$$ (6)

### 4.3 Details

The image size for input to the network in this study was set to 224*224. Both training and testing of the model were conducted in parallel on two NVIDIA Tesla V100 GPUs. The optimizer used was SGD (Stochastic Gradient Descent) with an initial learning rate of 0.01, momentum set to 0.9, and weight decay set to 1e^-4^. The batch size was set to 24, and the total number of epochs was set to 300.

### 4.4 Evaluation metrics

In this study, due to the significant disparity in the proportion of background and foreground information in skin image segmentation tasks, the model’s performance in supervised skin lesion segmentation was evaluated using several evaluation metrics: Dice coefficient (Dice), Intersection over union (Iou), Sensitivity (Sen), and Specificity (Spe). Higher values of Dice and Iou ratios indicate a higher overlap between the model’s segmentation and the actual segmentation, primarily measuring the model’s segmentation accuracy. Sen and Spe respectively represent the model’s recognition ability for positive and negative class samples. The formula is as follows7-10:

$$\mathrm{Iou}=\frac{TP}{TP+FP+FN}$$ (7)

$$\mathrm{Sen}=\frac{TP}{TP+FN}$$ (8)

$$\mathrm{Spe}=\frac{\mathrm{TN}}{\text{TN+FP}}$$ (9)

$$\mathrm{Dice}=\frac{\mathrm{2TP}}{\text{FP+2TP+FN}}$$ (10)

## 5. Experimental results

### 5.1 Ablation study

**Fig 4:**
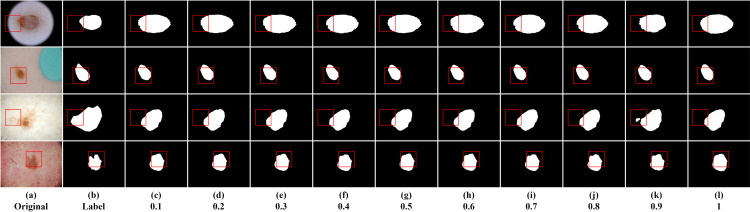
Segmentation instances for different sparsity ratios on ISIC2016. (a, b) are the original image and the ground truth mask, respectively; (c-l) are segmentation instances for different sparsity ratios.

**Fig 5:**
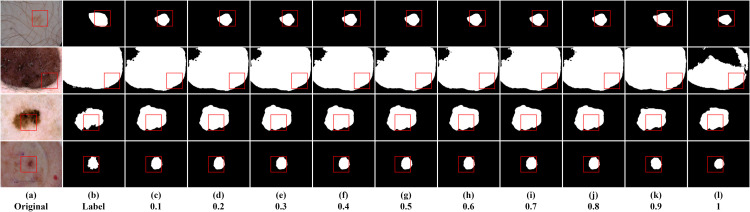
Segmentation instances for different sparsity ratios on ISIC2017. (a, b) are the original image and the ground truth mask, respectively; (c-l) are segmentation instances for different sparsity ratios.

**Fig 6:**
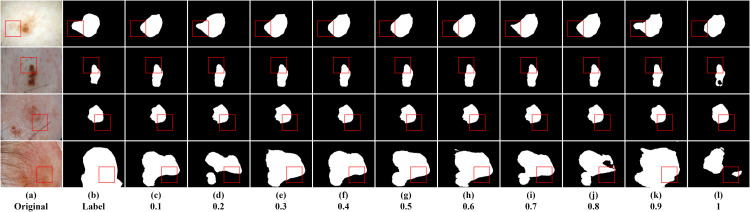
Segmentation instances for different sparsity ratios on ISIC2018. (a, b) are the original image and the ground truth mask, respectively; (c-l) are segmentation instances for different sparsity ratios.

In this section, we primarily investigated the impact of sparsity ratio on the model. The sparse matrix generated in the model follows a random distribution based on Bernoulli probability. Different probability values represent the proportion of information to be filtered out by default, where a higher probability value retains more information. This study examined the effect of sparsity ratios ranging from 0.1 to 1 on the experimental results, conducted on the ISIC2016, ISIC2017, and ISIC2018 datasets. Table 2 displays the results for ten sparsity ratios to identify the optimal solution. Higher values of Dice and Iou indicate higher segmentation accuracy. The optimal results are indicated in bold. Several segmentation instances are shown in Figs 4–6.

**Table 2:** Experimental results for different sparsity ratios.

Sparse Ratio	ISIC2016		ISIC2017		ISIC2018	
	Dice	Iou	Dice	Iou	Dice	Iou
0.1	0.912	0.849	0.850	0.764	0.896	0.826
0.2	0.913	0.851	0.850	0.764	0.894	0.823
0.3	0.912	0.849	0.848	0.763	0.896	0.826
0.4	0.910	0.847	0.850	0.765	0.898	0.827
0.5	0.911	0.848	0.850	0.764	0.894	0.823
0.6	0.912	0.849	0.850	0.764	0.896	0.825
0.7	0.911	0.849	0.849	0.764	0.895	0.826
0.8	0.911	0.849	0.850	0.764	0.895	0.824
0.9	0.914	0.851	0.857	0.775	0.898	0.830
1	0.906	0.841	0.834	0.745	0.886	0.815

From Table 2, we can infer the following, Firstly, when the last layer of the encoder employs the dynamic sparse attention mechanism to reduce the negative impact of redundant information in the feature maps on the results, it has a positive effect on the experimental results, regardless of the proportion of filtered information. This indicates that the dynamic sparse attention mechanism plays a beneficial role in enhancing the model’s performance. Secondly, the experimental results for sparsity ratios from 0.1 to 0.9 do not show significant differences. However, across the three datasets, the model’s performance reaches its optimal level when the sparsity ratio is 0.9. This suggests that 0.9 might be a suitable choice for the sparsity ratio as it performs well across different datasets. However, when the sparsity ratio is 1, meaning all information in the feature maps is retained, the model’s performance is the worst. This could be due to an overload of information leading to model overfitting or difficulty in handling complexity. Lastly, although the differences among other sparsity ratios are subtle, it can be observed that when the ratio of redundant information to effective information is close to 0.5, there is a slight negative impact on the segmentation results. This suggests the need to strike a balance between redundant and effective information when selecting the sparsity ratio to achieve the best segmentation performance. In summary, the dynamic sparse attention mechanism plays a vital role in improving segmentation performance, and selecting an appropriate sparsity ratio (0.9) can yield the best segmentation results across different datasets. Additionally, it’s important to note that a sparsity ratio close to 0.5 may have a minor negative effect on the segmentation results.

From the segmentation instance images, we can visually observe varying segmentation qualities. The segmentation result is closest to the ground truth mask when the sparsity ratio is 0.9. However, the segmentation performance is poorest when the sparsity ratio is 1. Taking the first row of ISIC2016 instance images as an example, the segmentation instance image is closest to the ground truth mask when the sparsity ratio is 0.9. Except for the segmentation instance with a sparsity ratio of 1, in the experiment, we found that a sparsity ratio of 0.5 resulted in relatively poor segmentation performance compared to other values. However, interestingly, when the sparsity ratio varied within the range of 0.1 to 0.9, the segmentation results symmetrically centered around 0.5 showed relatively consistent performance, indicating no significant differences in their performance. This suggests that the model’s performance might be more stable around lower and higher sparsity ratio values, while sparsity ratios around 0.5 may exhibit relatively poorer performance.

When most of the information is considered redundant, the network focuses more on effective information. However, as the sparsity ratio increases and the preserved feature information gradually approaches the filtered features, it starts to have a negative impact on the network. Nevertheless, when the preserved features are significantly more than the filtered features, the network can still make accurate judgments. Observing the third row of example images for ISIC2018, we can see that when the sparsity ratio is small, the boundaries of the segmentation instance images are clearer, while they appear more complete when the sparsity ratio is larger. This indicates that with a smaller sparsity ratio, the network emphasizes local information modeling but may sacrifice global information modeling, and vice versa. Therefore, in this study, we chose a sparsity ratio of 0.9 as the baseline for the network model, as it performed the best in terms of segmentation boundary accuracy and completeness in identifying lesion areas.

### 5.2 Comparative experiments

**Fig 7:**
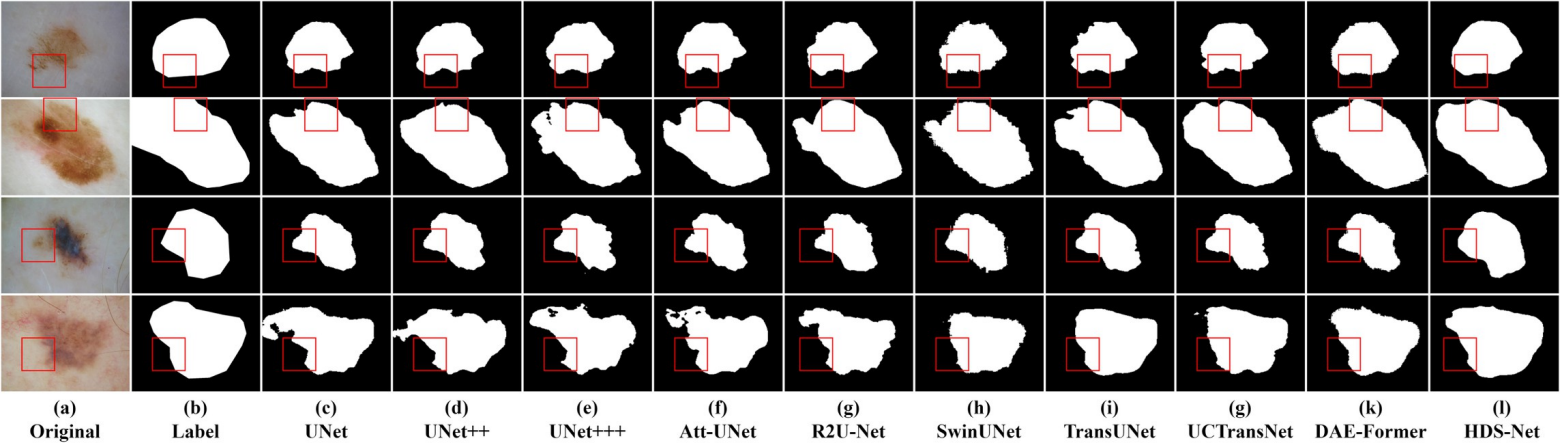
Segmentation instance examples for ISIC 2016 dataset.

**Fig 8:**
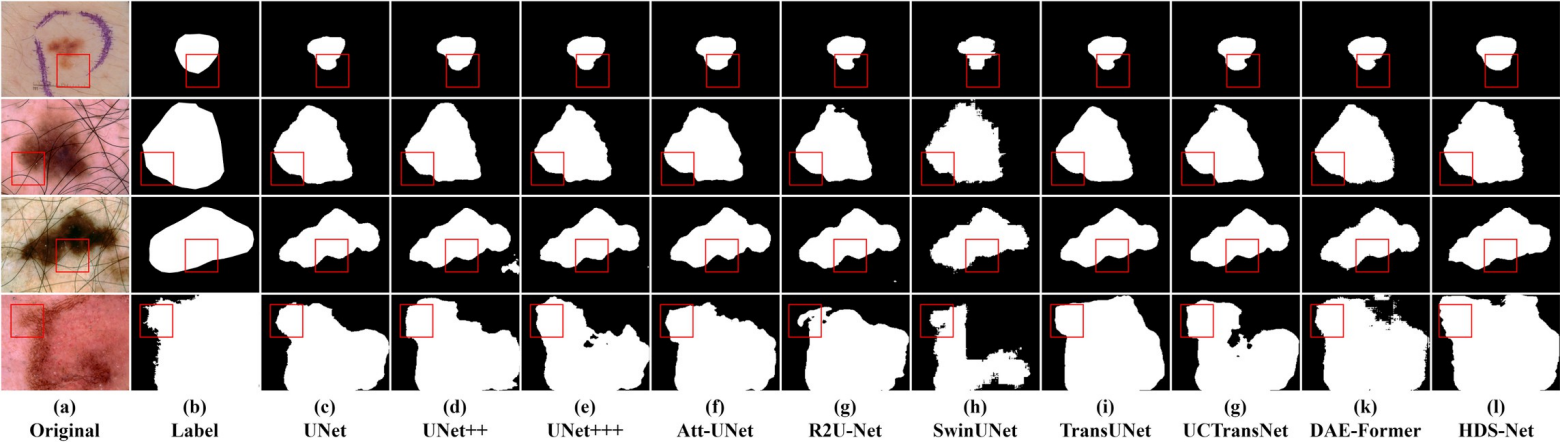
Segmentation instance examples for ISIC 2017 dataset.

**Fig 9:**
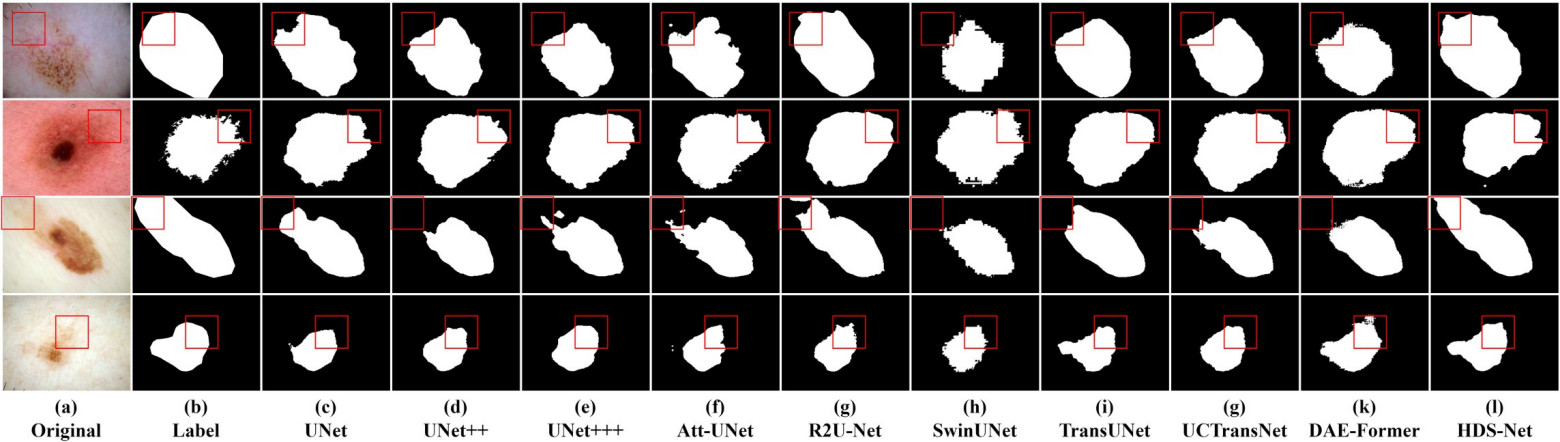
Segmentation instance examples for ISIC 2018 dataset.

In this section, we compared the proposed method with current state-of-the-art methods on three datasets. The results are shown in Tables 3–5, indicating that HDS-Net outperforms the other methods significantly and can be considered a reliable framework for skin lesion segmentation. Figs 7–9 respectively illustrate the comparative segmentation instance images on ISIC2016, ISIC2017, and ISIC2018 datasets with the SOTA models.

**Table 3:** Comparative experimental evaluation metrics for ISIC 2016.

Model	Dice	Iou	Sen	Spe
U-Net	0.899	0.833	0.913	0.959
U-Net ++	0.893	0.823	0.912	0.958
U-Net +++	0.891	0.82	0.905	0.963
Att-UNet	0.899	0.834	0.921	0.958
R2U-Net [41]	0.91	0.853	0.921	0.962
SwinUNet	0.883	0.821	0.913	.0.961
TransUNet	0.914	0.843	0.931	0.963
UCTransNet [42]	0.911	0.842	0.921	0.967
DAE-Former [43]	0.901	0.838	0.917	0.955
HDS-Net	0.914	0.851	0.937	0.959

**Table 4:** Comparative experimental evaluation metrics for ISIC 2017.

Model	Dice	Iou	Sen	Spe
U-Net	0.827	0.736	0.803	0.975
U-Net ++	0.829	0.743	0.816	0.971
U-Net +++	0.824	0.731	0.798	0.979
Att-UNet	0.834	0.746	0.813	0.969
R2U-Net	0.832	0.751	0.813	0.971
SwinUNet	0.812	0.713	0.832	0.953
TransUNet	0.821	0.734	0.781	0.983
UCTransNet	0.826	0.742	0.811	0.972
DAE-Former	0.813	0.726	0.797	0.972
HDS-Net	0.857	0.775	0.852	0.963

**Table 5:** Comparative experimental evaluation metrics for ISIC 2018.

Model	Dice	Iou	Sen	Spe
U-Net	0.891	0.811	0.893	0.969
U-Net ++	0.886	0.81	0.887	0.971
U-Net +++	0.876	0.793	0.893	0.964
Att-UNet	0.884	0.81	0.883	0.966
R2U-Net	0.894	0.841	0.899	0.976
SwinUNet	0.817	0.721	0.834	0.981
TransUNet	0.893	0.821	0.895	0.973
UCTransNet	0.886	0.817	0.891	0.982
DAE-Former	0.882	0.811	0.889	0.969
HDS-Net	0.898	0.83	0.894	0.974

Through Tables 3–5, it can be clearly observed that our proposed model performs satisfactorily in various evaluation metrics compared to traditional convolutional models and Transformer models. On the ISIC2016 dataset, HDS-Net outperforms most of the SOTA models. While HDS-Net’s Dice score is the same as TransUNet, our Sensitivity score is higher, indicating more accurate identification of lesion features in our network. On the ISIC2017 dataset, HDS-Net achieved significantly higher scores. Furthermore, on the ISIC2018 dataset, HDS-Net obtained the highest Dice score, although the Iou score is slightly lower than R2U-Net, it still outperforms other models. However, it is evident from the tables that the overall performance of all models on the ISIC2017 dataset is relatively poor. This is because the ISIC2017 dataset contains more complex lesion types and considerable background noise, posing a greater challenge for accurate segmentation. Therefore, it can be seen from the tables that HDS-Net demonstrates an advantage in both generalization performance and robustness.

However, a more intuitive way to evaluate segmentation quality is by observing segmentation instance images. From these images, it is evident that in skin images, when there is a significant difference between the lesion area and the normal area, both traditional convolutional neural networks and Transformer models can accurately segment these regions. But when the difference between the lesion area and the normal area is small, or when the boundary of the lesion area is blurry, there is a notable difference between traditional convolutional models and Transformer models.

For instance, in the third row of segmentation instance images for the ISIC2016 dataset, traditional convolutional networks like U-Net and U-Net++ exhibit sharper extraction of boundary information, resulting in shapes closer to the ground truth mask. However, they show a slight deficiency in segmentation completeness compared to models like TransUNet.

In the first row of segmentation instance images for the ISIC2017 dataset, it’s evident that models like TransUNet and SwinUNet produce segmentation images with incomplete segmentation due to the issue of blurry boundaries compared to the ground truth mask. In the third row of images, it is shown that when complex backgrounds are present in the image, both traditional convolutional and Transformer structures are prone to misclassification, resulting in incomplete or incorrect segmentation. However, HDS-Net’s recognition result is the closest to the ground truth mask.

In the third row of segmentation instance images for the ISIC2018 dataset, the boundaries in the original image are very blurry. The traditional convolutional structures recognize the blurry regions more than Transformer structures, but still, they cannot identify the lesion area completely. Among them, R2U-Net and TransUNet perform well in segmentation accuracy, but there is still a gap compared to the ground truth mask. On the other hand, HDS-Net’s segmentation instance images show significant advantages in both complex background and blurry boundary lesion images.

As shown in the Table 6, compared with the previous main networks, the proposed HDS-Net, while having the fewest parameters, is able to achieve excellent segmentation results, confirming that improved segmentation performance does not necessarily rely on a large number of parameters. This also verifies the effectiveness of the innovation points proposed in this paper.

**Table 6:** Parameters between different network architectures.

Model	Parameters(M)	FLOPs(G)
U-Net	34.54	50.18
Att-UNet	34.88	51.03
SwinUNet	27.17	5.92
TransUNet	105.32	24.63
HDS-Net	24.36	8.67

## 6. Conclusion

In the field of medical image analysis, computer-aided semantic segmentation plays a crucial role in improving and expediting the diagnostic process. This study adopts a symmetric encoder-decoder based on the U-shaped network architecture. By using a hybrid encoder, we can better extract features and establish connections between global and local information. At the bottom of the encoder, a dynamic sparse module is designed, which utilizes two learnable memory units to establish potential connections among different samples. Additionally, by controlling the sparsity ratio of the sparse matrix to suppress the negative impact of redundant information, we explore the effect of the ratio of preserved and filtered information at the last layer of the encoder on experimental results. Furthermore, this paper enhances the modeling capability of local information through element-wise multiplication, which helps the model better identify detailed features. This effectively addresses some challenges in skin lesion segmentation, such as indistinct boundaries, differences among different lesion characteristics, as well as the complexity of lesions and redundancy in background information.

The key to the dynamic sparse attention mechanism lies in introducing randomness through a Bernoulli masking matrix to achieve dynamic focus on different regions of the image. However, the introduction of such randomness may bring about some potential issues. Specifically, the presence of randomness can lead to the model overfitting to noise during the training process. Since the model may produce different outputs for the same input due to this randomness, it becomes more susceptible to the influence of noise and outliers in the training data, thereby reducing the model’s stability.

Furthermore, if there is class imbalance in the training data, where one category or lesion dominates the dataset, the model may learn features primarily associated with the dominant category, resulting in suboptimal segmentation performance for other lesion types. This is because, when faced with the dominant category, the model, influenced by randomness, may tend to prioritize learning features that are more prominent in the training data, while relatively less frequent categories may be neglected.

Taking these issues into consideration, we will adopt a dynamic adaptive strategy to introduce randomness in the model training process in future work, aiming to balance the model’s ability to fit and generalize data. Initially, a higher degree of randomness is introduced during the early stages of model learning, promoting a more comprehensive exploration of different features and solution spaces to facilitate a more thorough learning of the training data. As training progresses, the introduction of randomness is gradually reduced or avoided altogether to ensure the model focuses more on learning the true data distribution and patterns, thereby reducing sensitivity to noise in the training data.
